# Combining systemic and stereotactic MEMRI to detect the correlation between gliosis and neuronal connective pathway at the chronic stage after stroke

**DOI:** 10.1186/s12974-016-0622-7

**Published:** 2016-06-18

**Authors:** Xiao-zhu Hao, Le-kang Yin, Xiao-xue Zhang, Jia-qi Tian, Chan-chan Li, Xiao-yuan Feng, Min Jiang, Yan-mei Yang

**Affiliations:** Department of Radiology, Huashan Hospital, Fudan University, Shanghai, 200040 China; Institutes of Science and State Key Laboratory of Medical Neurobiology, Fudan University, Shanghai, 200032 China

**Keywords:** Stroke, Neuroinflammation, Neuronal connective pathway, MEMRI, Astrogliosis, Microglia/macrophage

## Abstract

**Background:**

The early dysfunction and subsequent recovery after stroke, characterized by the destruction and remodeling of connective pathways between cortex and subcortical regions, is associated with neuroinflammation. As major components of the inflammatory process, reactive astrocytes have double-edged effects on pathological progression. The temporal patterns of astrocyte and neuronal pathway activity can be revealed by systemic and stereotactic manganese-enhanced magnetic resonance imaging (MEMRI), respectively. In the present study, we aimed to detect an association between astrocyte activity and recovery of neuronal connective pathways by combining systemic with stereotactic MEMRI.

**Methods:**

Fifty adult rats, divided into two groups, underwent a 60-min occlusion of the middle cerebral artery. The groups were given either a systemic administration or stereotactic injection of MnCl_2_ at 1, 3, 7, and 14 days after stroke and underwent MRI 4 and 2 days later, respectively. Immunofluorescence (IF) of group 1 was conducted to corroborate the results. Repetitive behavioral testing was also performed with all rats at 1, 3, 7, and 14 days to obtain a functional score.

**Results:**

Ring- or crescent-shaped enhancements formed in the striatal peri-infarct regions (STR) at 11 and 18 days. This was concurrent with the activity of glial fibrillary acidic protein (GFAP)-positive astrocytes, which mainly localized at the peri-infarct region and significantly increased in number at 11 and 18 days after stroke. Microglia/macrophages, detected by IF, mainly localized in the lesion core, rather than in the region of enhancement. The ipsilateral substantia nigra (SN) revealed Mn-related signal enhancement reduction and subsequent signs of the recovery process at 3 to 5 days and 9 to 16 days, respectively. Behavioral testing showed that sensorimotor functions were initially disturbed, but subsequently recovered at 7 and 14 days.

**Conclusions:**

We found a positive temporal correlation between astrogliosis and the recovery of neuronal connective pathways at the chronic stage by using the in vivo method of MEMRI. Our results highlighted the potential contribution of astrocytes to the neuronal recovery of these connective pathways.

**Electronic supplementary material:**

The online version of this article (doi:10.1186/s12974-016-0622-7) contains supplementary material, which is available to authorized users.

## Background

Stroke is the second leading cause of disability and mortality worldwide [1–3]. The compromised blood-brain barrier (BBB) after stroke facilitates neuroinflammation, which has long-standing effects on brain function and a clear linkage to the degree of brain damage. Axonal disconnection and breakdown of axonal cytoskeletal components resulting from ischemia may account for the dysfunction of remote regions connected to the cortex. Subsequent recovery may be associated with the restoration and reorganization of the connective pathway [4].

As major components of inflammation, reactive astrocytes, which initiate structural and functional modulation after ischemia, have been studied intensively [5]. With the advance of magnetic resonance imaging (MRI), it has become possible to longitudinally monitor the dynamic changes of cellular activity by using a contrast agent. Manganese, as the MR contrast agent, has been widely used to track changes in neuronal activity [4, 6–8], and a recent study showed that Mn^2+^ could aid in the visualization of reactive astrogliosis with manganese-enhanced magnetic resonance imaging (MEMRI) [9]. However, the time-dependent evolution of inflammatory cellular responses and structural changes of neuronal connective pathways still need clarification. Furthermore, in vivo visualization of the association between specific cellular responses and neuronal connective pathway repair needs investigation in order to provide more preclinical information for diagnosis, treatment, and recovery [10]. In this study, manganese was used to trace cellular and neuronal temporal alterations and their associations after stroke by systemic and stereotactic MEMRI. With further corroboration by immunofluorescence (IF), the specific cellular response, which was more associated with neuronal remodeling of the connective pathway, was outlined. The primary objective of our study was to longitudinally observe the temporal dynamic patterns of astrogliosis and neuronal connective pathway changes in vivo, as well as the associations between these processes after transient cerebral ischemia.

## Methods

### Animals

**Fig. 1:**
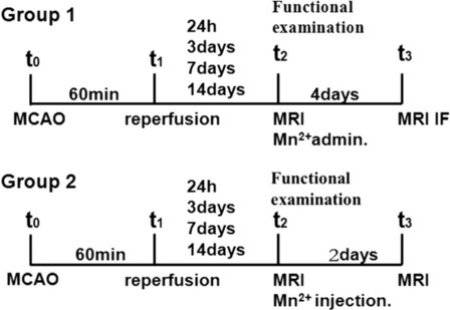
Experimental procedure. The left middle cerebral artery was occluded for 60 min for all of the MCAO subgroups. MRI data were acquired to check occlusion, brain edema, and intracerebral bleeding of the left brain hemisphere 24 h later for the control and day 1 MCAO subgroups, 3 days later for the day 3 MCAO subgroup, 7 days later for the day 7 MCAO subgroup, and 14 days later for the day 14 MCAO subgroup. Functional examination was repetitively performed at the same time points prior to MRI. MRI data were acquired again exactly 4 and 2 days later for group 1 and group 2, respectively. Rats of group 1 were sacrificed for immunofluorescence staining

All animal procedures performed in this study were approved by the Fudan University Institutional Animal Care and Use Committee. A total of 50 male Sprague Dawley rats (280 to 300 g) were subjected to 60 min of MCAO and were then divided into two experimental groups. Rats in group 1 (*n* = 25) were subjected to systemic administration of MnCl2 through their tail veins, while rats in group 2 (*n* = 25) were subjected to stereotactic injection of MnCl2 into the peri-lesional cortex. In each of the two groups, rats were divided into five subgroups: control (*n* = 5), day 1 (*n* = 5), day 3 (*n* = 5), day 7 (*n* = 5), and day 14 (*n* = 5). Figure 1 shows the brief procedures for groups 1 and 2 (Fig. 1).

### Experimental procedures

Rats were anesthetized with an intraperitoneal injection of 10 % chloral hydrate under spontaneous inspiration, and the body temperature was continuously monitored at 37 ± 0.5 °C during the surgical procedures. For all rats undergoing MCAO, the left middle cerebral artery (MCA) was occluded for 60 min. In detail, rats were immobilized by a tooth holder and with binding of all limbs, followed by the insertion of a 4.0 silicon-coated polypropylene suture into the left internal carotid artery (ICA) through the external carotid artery (ECA) and common carotid artery (CCA) to block blood flow to the MCA. After 60 min, the filament was withdrawn from the ICA to allow reperfusion. Two control subgroups experienced identical operations but without MCA occlusion. MRI data were acquired under anesthetized circumstances to examine brain edema and intracerebral bleeding of the left brain hemisphere 24 h later for the control and day 1 MCAO subgroups, 3 days later for the day 3 MCAO subgroup, 7 days later for the day 7 MCAO subgroup, and 14 days later for the day 14 MCAO subgroup. Additionally, a functional examination was performed repetitively at the same time points prior to MRI. Immediately after MRI scanning, group 1 received 50 mM of a MnCl2 solution (267.9 μmol/kg) delivered via syringe at a rate of 1.2 ml/h through the tail vein. Group 2 received 0.2 μl of 1 M MnCl2, injected with a 2.0-μl Hamilton syringe at a rate of 0.05 μl/min via the burr hole. MRI data were acquired again 4 days later for group 1 and 2 days later for group 2. Finally, rats of group 1 were sacrificed for immunofluorescence staining by decapitation under deep anesthesia.

### Functional examination

Animals were subjected to behavioral tests to assess sensorimotor function at 1, 3, 7, and 14 days after stroke. We scored motor, sensory, and tactile tests according to a neurological scale of 0 to 20 points, with 20 representing maximal deficit [11].

### Magnetic resonance imaging

Prior to MRI, animals were anesthetized by the same procedure as described for the MCAO model (see above). The body temperature was continuously maintained at 37 ± 0.5 °C, and blood oxygen saturation and heart rate were monitored during MRI procedures. The MRI measurements were performed on a 3.0-T horizontal magnet (Discovery MR750, GE Medical Systems, Milwaukee, WI) with a 60-mm-diameter gradient coil (Magtron Inc., Jiangyin, China).

T_2_-weighted MR images were first obtained by fast spin-echo sequence with the following acquisition parameters: Repetition time (TR)/Echo time (TE) = 4000 ms/96 ms, scan time = 3 min, Field of view (FOV) = 6 cm × 6 cm, matrix = 256 × 256, slice thickness (ST) = 1.8 mm, spatial resolution = 0.24 × 0.24 × 1.8 mm^3^, inter-slice distance = 2 mm, number of slices = 15, and number of averages (NA) = 2. Three-dimensional T_1_-weighted MR images were acquired by a gradient-recalled echo sequence with the following acquisition parameters: TR/TE = 12 ms/6 ms, scan time = 3.09 min, FOV = 7 cm × 7 cm, matrix = 256 × 256, ST = 1 mm, spatial resolution = 0.27 × 0.27 × 1 mm^3^, inter-slice distance = 1 mm, number of slices = 60, and NA = 1, flip angle = 15°.

### Manganese-enhanced MRIs

All procedures were performed under deep anesthesia as described for the MCAO model (see above) with the body temperature, blood oxygen saturation, and heart rate monitored at normal levels. For group 1, rats were administered isotonic MnCl_2_ · 4H_2_O at a concentration of 50 mM (267.9 μmol/kg) through the tail vein at the rate of 1.2 ml/h. This can be tolerated by rats with minimal death rate. For group 2, rats were placed in a stereotactic holder and immobilized with earplugs and a tooth holder. A burr hole was drilled in the skull 0.5 mm anterior and 1.2 mm lateral to the bregma and above the sensorimotor cortex according to the Paxinos and Watson atlas (1998) [12]. MnCl_2_ (0.2 μl 1 M) was injected with a 2.0-μl Hamilton syringe at a rate of 0.05 μl/min. After injection, the needle was left in place for 3 min to prevent leakage. After systemic administration and stereotactic injection of MnCl_2_ to group 1 and group 2, respectively, rats were returned to their cages with sufficient food and water, and MR images of each group were acquired exactly 4 and 2 days later, respectively, as described above.

### Immunofluorescence

Removed brain samples from group 1 rats were post-fixed in 4 % paraformaldehyde for 24 h and then were vitrified in 20 % and 30 % sucrose solutions for 24 h and 3 days, respectively. Coronal brain sections (30 μm) of all rats were obtained using a cryostat (RM2135, Leica) and were preserved in cryoprotectant at −20 °C until further use. Immunohistochemistry was performed on two cryosections of each rat, from approximately 1.70 to −4.80 mm relative to the bregma, according to the Paxinos and Watson atlas (1998) [12]. One section from each rat was double-stained with anti-glial fibrillary acidic protein (GFAP) (1:300, GeneTex, Texas, USA) and NeuN (1:250, Biosensis, Thebarton, Australia). Another section from each rat was stained with anti-CD11b (1:200, AbD Serotec, Kidlington, UK). In detail, sections were washed three times with phosphate buffered saline (PBS, pH = 7.4). Sections were then blocked from non-specific binding with 10 % normal donkey serum in PBS containing 0.3 % Trition-X-100 (Sigma-Aldrich, St. Louis, MO, USA) for 2 h at room temperature. They were then incubated with primary antibodies overnight at 4 °C. For double-stained sections, tissues were then washed and incubated for 2 h at room temperature with corresponding fluorochromated secondary antibodies (each 1:200, donkey anti-mouse Alexa Fluor 488 and donkey anti-rabbit Alexa Fluor 568, Life Technologies, Carlsbad, CA, USA). After washing, sections were incubated with DAPI (1:1000, Sigma-Aldrich, St. Louis, MO, USA) for 5 min. For single-stained sections, tissues were washed and incubated with a secondary antibody (1:200, donkey anti-mouse Alexa Fluor 488, Life Technologies, Carlsbad, CA, USA). All sections were washed and had coverslips placed with mounting media. Sections from different groups were processed in the same batches to minimize staining variability.

### Image processing and quantitative analysis

To obtain magnetic resonance images, we placed the manually drawn regions of interest (ROIs) of striatum (STR) and substantia nigra (SN) in both hemispheres onto slices as shown in Figs. 4 and 5. Semi-quantitative signals of ROIs were calculated from the T_2_-weighted MR images and T_1_-weighted MR images and were normalized by the contralateral site equivalent to the ROIs for analysis. T2 MR images were used to show the location of striatal ischemic lesions, and images with no striatal T2 lesions or with very large hyperintense lesions extending to the contralateral hemisphere were excluded from analysis.

**Fig. 2:**
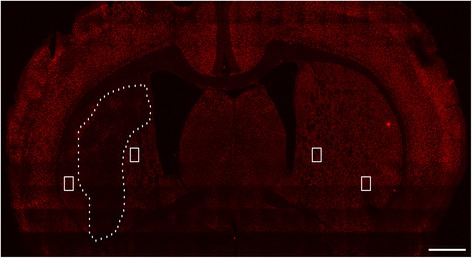
Representative brain section stained for NeuN showing the regions of interest used for GFAP/NeuN analysis. *Scale bar* = 1 mm

To obtain GFAP/NeuN immunofluorescence staining images, sections were scanned with the ×20 primary objective of a Vslide scanning microscope (Nikon, Chiyoda, Tokyo, Japan) with filter sets for DAPI (EX350/50-EM470/40), FITC (EX493/16-EM527/30), and FRITC (550,620). An initial capture was stitched into Vslide software to create the integrated images of the whole brain from the representative brain sections, showing the delineation of the ROI for facilitating analysis. The ROI used for analysis was drawn on the representative brain section stained for NeuN (Fig. 2). Images of the contralateral site equivalent to the location of the above ROIs were also obtained. Images of these areas were acquired with a resolution of 1024 × 1024 pixels using constant values for laser power, pinhole, digital gain, offset, and scan speed. For CD11b immunofluorescence staining images, sections of an animal’s ipsilateral hemisphere were first scanned with the 10× objective using a fluorescence microscope (Olympus PX51, Olympus Corporation, Shinjuku-ku, Japan) and were then “photomerged” using Adobe Photoshop 3.0 software to show the ROI for analysis of microglia (Fig. 7). Slices of the ischemic cores were obtained from all rats for quantitative analysis. Enlarged images of ROIs were scanned with a ×20 objective lens.

The acquired images were further analyzed using Image J (National Institutes of Health, Bethesda, MD, USA). For analysis and quantification, we used the mean staining density of immunofluorescence, which can proportionally reflect both the cell number and level of the targeted protein. The intensity of fluorescence was calculated as the mean of the intensity from the imaged sections and was normalized by the contralateral site.

### Statistical analysis

One-way analysis of variance (ANOVA) was performed for multiple group comparisons with post hoc least-significant difference (LSD) tests performed for each of the two groups. The correlation between histological changes and MEMRI was assessed using Pearson Product correlation analysis. In all statistical tests, data were presented as the mean ± SD, and differences were considered significant when *P* < 0.05. Statistical analysis was performed using SPSS (19.0).

## Results

### Changes of striatum in T_2_-weighted MR images after MCAO

**Fig. 3:**
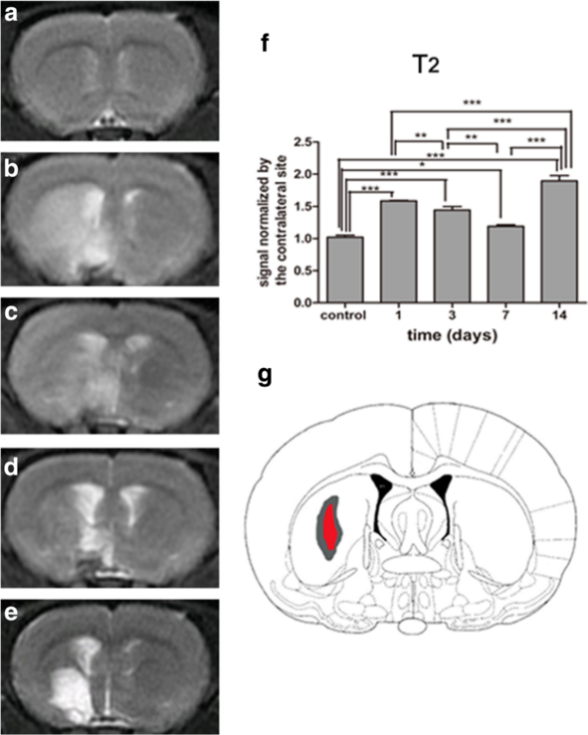
Longitudinal observation of T2 for STR of group 1 after MCAO. T2 MR images of control (**a**), day 1 (**b**), day 3 (**c**), day 7 (**d**), and day 14 (**e**). Comparisons of T2 signal between subgroups (**f**). ^***^ *P* < 0.001 versus day 14 and control, day 1, day 3, and day 7. ^*^ *P* < 0.05 versus day 7 and control, ^**^ *P* < 0.01 versus day 7 and day 3, ^***^ *P* < 0.001 versus day 3 and control, ^**^ *P* < 0.01 versus day 3 and day1, ^***^ *P* < 0.001 versus day 1 and control. Anatomical reference showing the ROI (**g**)

The location of the ipsilateral ischemic lesion was characterized by a prolonged T2 signal. During our observation time, the T2 signal was significantly elevated at 1 and 3 days after MCAO compared to control rats (*P* = 0.000) (Fig. 3a–c). It then declined at 7 days (Fig. 3d) and significantly rose again compared to all previous time points when necrosis occurred 14 days after reperfusion (*P* = 0.000) (Fig. 3e).

### MEMRI detection of enhancement after MCAO

To investigate the dose, concentration, and time dependence of brain enhancement, we firstly performed systemic and stereotactic MEMRI with normal rats and stroke models at different time points after administration of MnCl_2_ with various doses and concentrations (Additional file 1: Figure S1), and found that systemic MEMRI of STR had the best contrast 4 days after MnCl_2_ administration (50 mM, 267.9 μmol/kg), while stereotactic MEMRI of SN had the best contrast 2 days after MnCl_2_ injection (0.2 μl 1 M).

**Fig. 4:**
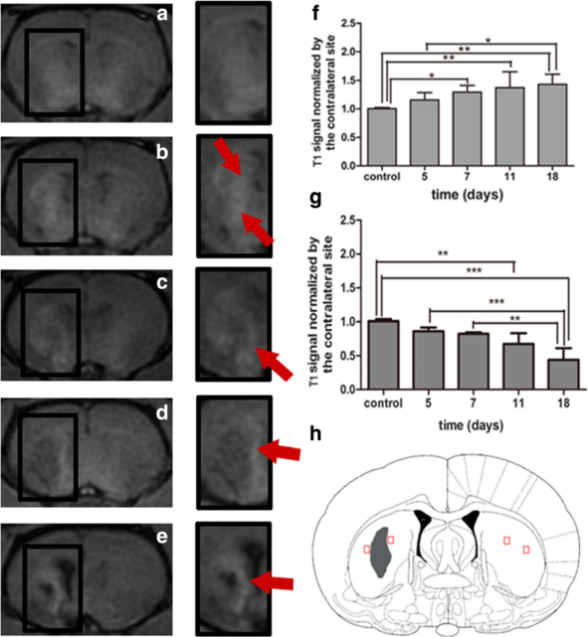
Longitudinal observation of manganese-enhanced MRI for STR of group 1 after MCAO. T1 MR images of ipsilateral hemisphere and magnification of STR of the control (**a**), day 5 (**b**), day 7 (**c**), day 11 (**d**), and day 18 (**e**). *Red arrows* of the enlarged images show the ring- or crescent-shaped manganese enhancement surrounding the ischemic core. Semi-quantification of T1 signal of the peri-infarct region of STR in the ipsilateral hemisphere (**f**). ^*^ *P* < 0.05 versus day 7 and the control. ^**^ *P* < 0.01 versus day 11 and control. ^**^ *P* < 0.01 versus day 18 and control. ^*^ *P* < 0.05 versus day 18 and day 5. Semi-quantification of T1 signal of the lesion core of STR in the ipsilateral hemisphere (**g**). ^**^ *P* < 0.01 versus day 11 and control. *P* < 0.001 versus day 18 and control. ***P < 0.001 versus day 18 and day 5. **P < 0.01 versus day 18 and day 7. Anatomical reference showing the ROI (**h**)

Then for group 1, slight MEMRI enhancement was observed across the bilateral STR of control rats 4 days after Mn^2+^ administration (Fig. 4a). Mn-related signal enhancement spread to the peri-infarct region at 5 days after MCAO, and there was no enhancement in the unaffected brain tissue (Fig. 4b). At 7 days after MCAO, enhancement of the peri-infarct region statistically increased compared to the control group (*P* = 0.038) (Fig. 4c). At 11 and 18 days after MCAO, ring- or crescent-shaped enhancements, both statistically significant compared with control rats (*P* = 0.01, *P* = 0.004), were clearly observed in the peripheral region of the ischemic core (Fig. 4d, e). There was no enhancement within the lesion core after stroke, and the T1 signal gradually decreased in our observation time (Fig. 4g).

**Fig. 5:**
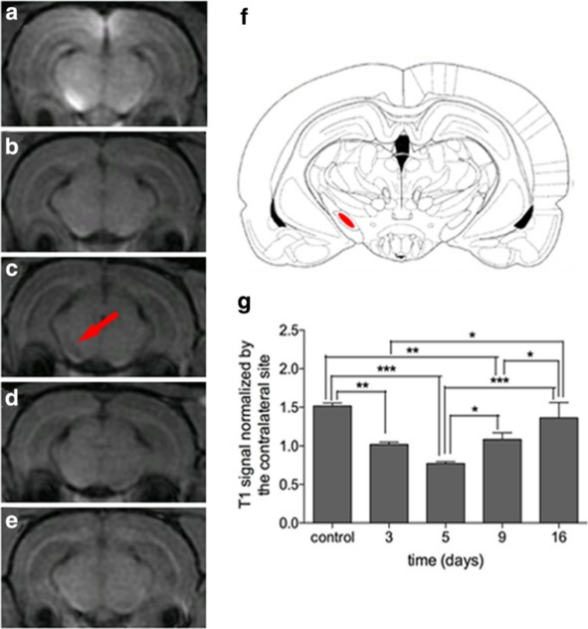
Longitudinal observation of manganese-enhanced MRI for SN of group 2 after MCAO. T1 MR images of the control (**a**), day 3 (**b**), day 5 (**c**), day 9 (**d**), and day 16 (**e**). *Red arrow* shows signal reduction of SN. Anatomical reference showing the ROI (**f**). Semi-quantification of T1 signal of SN in the ipsilateral hemisphere (**g**). ^**^ *P* < 0.01 versus day 3 and the control. ^**^ *P* < 0.05 versus day 5 and control. ^**^ *P* < 0.01 versus day 9 and control. *P < 0.05 versus day 9 and day 5. ^***^ *P* < 0.05 versus day 16 and day 5. ^*^ *P* < 0.05 versus day 16 and day 9. ^*^ *P* < 0.05 versus day 16 and day 3

And for group 2, Mn-related enhancement was clearly visualized in the SN, ipsilateral to the injection site, among control rats (Fig. 5a). A statistically significant decline of enhancement was observed at 3 days after stroke (*P* = 0.004) (Fig. 5b). At 5 days after MCAO, the enhancement further declined compared to the control group (*P* = 0.000) (Fig. 5c). Enhancement recovered slightly at 9 days compared to 5 days after ischemia (*P* = 0.019), although it was still lower overall than in control rats (*P* = 0.003) (Fig. 5d). Enhancement recovered further at 16 days after ischemia compared to 3 days (*P* = 0.041), 5 days (*P* = 0.000), and 9 days (*P* = 0.041), respectively, and there was no significant difference at 16 days when compared to control rats (Fig. 5e).

### Immunofluorescent stainings

#### Reactive astrocytosis and neuronal activity

**Fig. 6:**
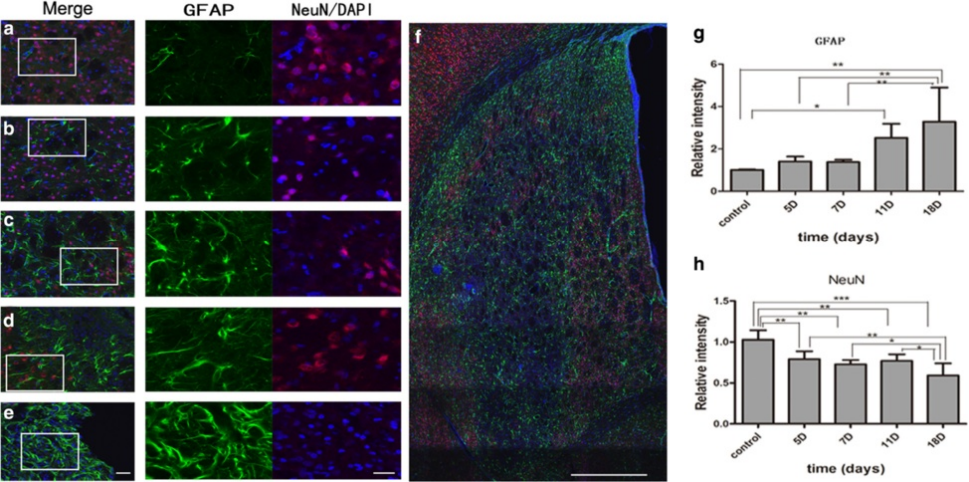
Glial (GFAP+) and neuronal nuclei (NeuN+) activity after MCAO. Double immunostaining of GFAP/NeuN at edges of lesion core of control (**a**), day 5 (**b**), day 7 (**c**), day 11 (**d**), and day 18 (**e**). The corresponding *right panels* present ×20 magnification images of GFAP and NeuN/DAPI. The “photomerged” image shows the entire focus of GFAP/NeuN in STR of the ipsilateral hemisphere (**f**). Quantification of GFAP fluorescence intensity in the ischemic hemisphere over reperfusion courses (**g**). ^*^ *P* < 0.05 versus day 11 and the control. ^**^ *P* < 0.01 versus day 18 and control, day 5, day 7. Quantification of NeuN fluorescence intensity in the same area of GFAP over reperfusion courses (**h**). ^**^ *P* < 0.01 versus day 5 and control. ^**^ *P* < 0.01 versus day 7 and control. ^**^ *P* < 0.01 versus day 11 and control. ^***^ *P* < 0.001 versus day 18 and control. ^**^ *P* < 0.01 versus day 18 and day 5. ^*^ *P* < 0.05 versus day 18 and day 7, day 11. *Scale bars* = 50 and 20 μm

GFAP and NeuN were used to study reactive astrocytosis and neuronal activity within the peri-lesional area as delineated by the presented brain section (Fig. 2). On day 5 and day 7 (Fig. 6b, c), a relatively small number of GFAP (+) cells could be observed within the peri-lesional area. The cell load significantly increased at 11 days (*P* = 0.032) and peaked at 18 days (*P* = 0.003) with more and longer extending processes compared to control rats (Fig. 6d, e). However, neuronal cells decreased gradually in number at 5 days (*P* = 0.01), 7 days (*P* = 0.002), 11 days (*P* = 0.006) and 18 days (*P* = 0.000) after ischemia compared to control rats (Fig. 6b–e).

### Reactive microgliosis

**Fig. 7:**
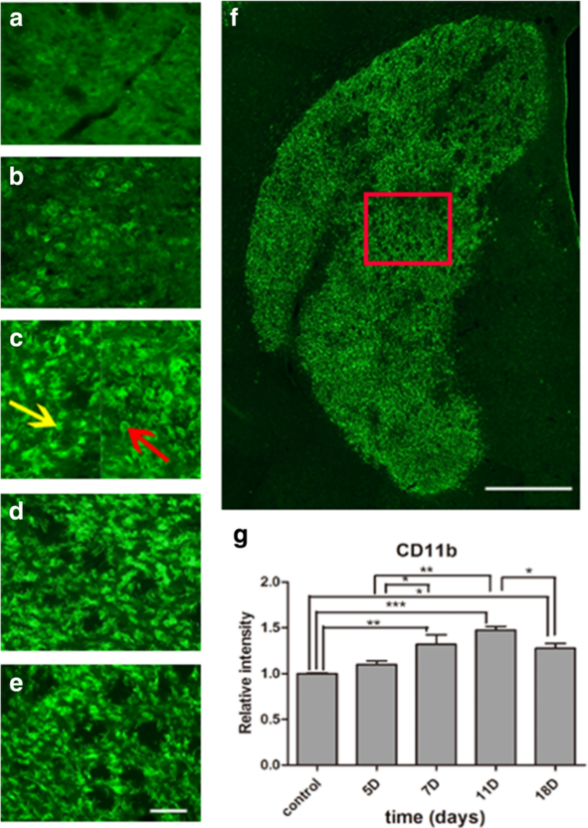
Microglia/macrophage activity after MCAO. Immunostaining of CD11b at the lesion core of the control (**a**), day 5 (**b**), day 7 (**c**), day 11 (**d**), and day 18 (**f**). The *red arrow* shows the round-shaped microglia/macrophages in the lesion core, and the *yellow arrow* shows the microglia/macrophages with extending processes adjacent to the peri-lesional area. The “photomerged” image shows the ROI of the lesion core, indicated by the box in *red* (**f**). Quantification of CD11b fluorescence intensity in the ischemic hemisphere during reperfusion (**g**). ^**^ *P* < 0.01 versus day 7 and control. ^***^ *P* < 0.001 versus day 11 and control. ^*^ *P* < 0.05 versus day 18 and control. ^*^ *P* < 0.05 versus day 7 and day 5. ^**^ *P* < 0.01 versus day 11 and day 5. ^*^ *P* < 0.01 versus day 18 and day 11. *Scale bars* = 50 and 500 μm

To evaluate the specific location and dynamic pattern of microglia, we performed immunohistochemistry with antibodies against CD11b. CD11b, one of the most commonly used surface markers for immunostaining microglia/macrophages, is upregulated after microglia/macrophage activation. There is no specific antibody representing microglia because microglia and macrophages tend to express very common cell markers [10]. Therefore, CD11b (+) cells were referred to as microglia/macrophages. On day 5, round CD11b (+) cells appeared throughout the entire lesion core, although there was no statistically significant difference compared to the control group (Fig. 7a, b). On day 7, cells with extending processes began to appear. Round CD11b (+) cells (red arrow) were mainly located in the lesion core, while cells with extending processes (yellow arrow) were mainly located adjacent to the peri-lesional area. The total relative intensity was increased compared to control rats (*P* = 0.003) (Fig. 7c). On day 11, though round CD11b (+) cells disappeared, a significant increase of total intensity of microglia/macrophage was established (*P* = 0.000) (Fig. 7d). By day 18, microglia activity decreased, but levels were still significantly higher than in the control group (*P* = 0.008) (Fig. 7e).

### Comparison between MEMRIs and immunofluorescent stainings

**Fig. 8:**
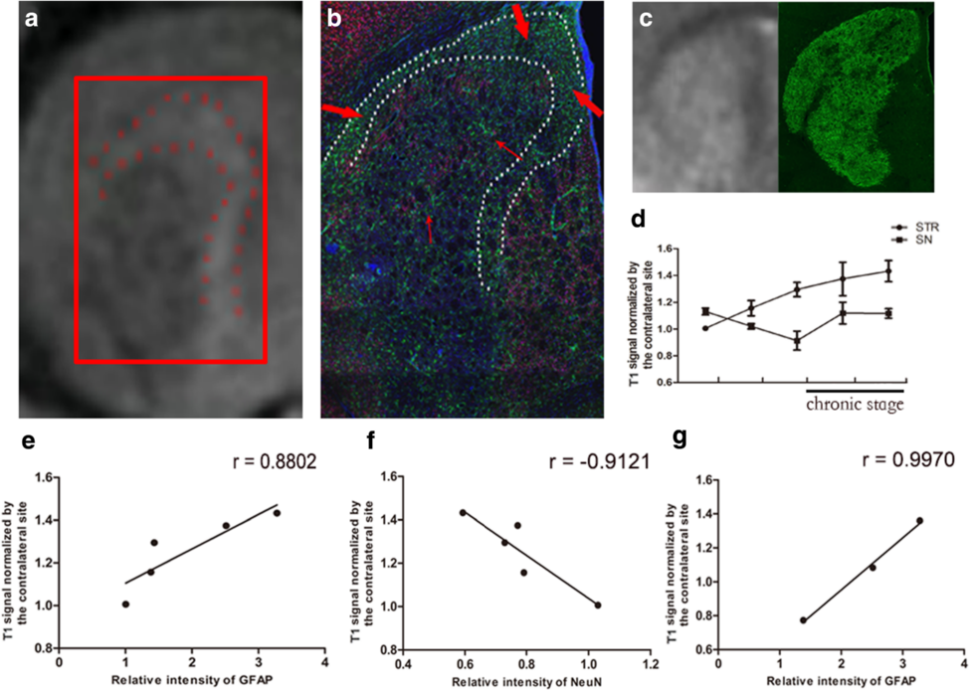
Comparison of MEMRI and IF. T_1_-weighted image shows the ring- or crescent-shaped enhancement on MEMRI (**a**, area located in the *red dash line*), concordant with the GFAP (+) cells observed in the area between the lateral ventricle and ischemic region (**b**, *thick red arrow*). The scattered enhancement in the lesion core was concordant with the GFAP (+) cells sparsely located inside the lesion (**b**, *thin red arrow*). T_2_-weighted MR image of the same animal shows the necrotic core (**c**, *left*). CD11b (+) immunostaining shows the area where microglia/macrophages mainly localized (**c**, *right*), concordant with the T_2_-weighted MR images. The *graph line* shows the same changes at later stages after reperfusion (**d**). Analysis of positive correlation between astrocyte activation and systemic MEMRI enhancement (**e**, *r* = 0.8802, *P* = 0.0489). Analysis of negative correlation between neuronal damage and systemic MEMRI enhancement (**f**, r = −0.9121, *P* = 0.0309). Analysis of positive correlation between astrocyte activation and stereotactic MEMRI enhancement (**g**, *r* = 0.9970, *P* = 0.0497). *Scale bars* = 500 μm

To observe the total cell density changes of the lesion area following cerebral ischemia showed in the T2 MR images, we analyzed the HE staining images (Additional file 2: Figure S2), which indicated that there were both increasing proliferative cells within and surrounding the ischemic lesion. Then, by the IF analysis, we have confirmed that both astrocytes and microglia/macrophage were increased during our observation time, and the former mainly located surrounding the lesion area while the latter mainly located within the infarct zone (Fig. 8b, c). Furthermore, both the location and time dependence of GFAP (+) cells of STR were in agreement with MEMRI on the same area (Fig. 8a, b, e), which means that MEMRI can represent the spatial and temporal evolution of astrocytes. To detect the causal relationship between astrocyte activation and recovery of SN, we performed the correlation analysis between relative intensity of astrocytes and MEMRI on SN, which showed the positive correlation between each other (*r* = 0.9970, *P* = 0.0497) (Fig. 8g). All these results explained the same temporal evolution of MEMRI on STR and SN (Fig. 8d), which implied the potential contribution of reactive astrocytes to the recovery of SN.

### Functional status

**Fig. 9:**
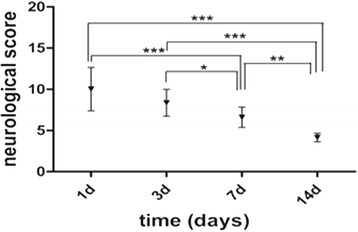
Neurologic score. ^***^ *P* < 0.001 versus day 7 and day 1. ^*^ *P* < 0.05 versus day 7 and day 3. ^***^ *P* < 0.001versus day 14 and day 1, day 3. ^**^ *P* < 0.01versus day 14 and day 7

Figure 9 shows changes in neurologic score as a function of time after stroke. Rats showed substantial functional deficits at 1 and 3 days after stroke. Functional recovery was reflected by statistically significant improvements of the neurologic score on day 7 compared to day 1 (*P* = 0.000) and day 3 (*P* = 0.042). By day 14 after stroke, rats showed significantly more improvement compared to day 1 (*P* = 0.000), day 3 (*P* = 0.000) and day 7 (*P* = 0.001).

## Discussion

In this study, the MCAO model with transient cerebral ischemia was used to demonstrate the post-ischemia neuroinflammatory response of the neuronal connective pathway between the cortex and subcortical area. First, we detected reactive striatal gliosis by systemic MEMRI, with in vivo administration of Mn^2+^ and immunohistochemistry. The results demonstrated that ischemia could promote the migration and proliferation of astrocytes. Mn-related signal enhancement first appeared throughout the entire lesion at 5 days after ischemia and then spread to the peri-infarct area, appearing as ring- or crescent-shaped enhancements. This was corroborated by IF analysis, as GFAP (+) cells were seen to spread to the border zone at later stages and were mainly localized to the area between the ventricle and STR. At the same time, stereotactic MEMRI of ipsilateral SN showed that signal enhancement declined at 3 and 5 days and recovered at 9 and 16 days after stroke, showing the dysfunction and subsequent recovery of the connective pathway from insult. The positive correlation of the temporal dynamics of astrogliosis and recovery of the connective pathway at the chronic stage demonstrated their underlying association with each other. Simultaneously, the resident microglia were activated to change from a round to amoeboid shape. This might also contribute to the recovery of the connective pathway and may need further study. In contrast to GFAP (+) cells, NeuN (+) cells of the selected ROI decreased in all time points.

Recently, the impact of inflammation on neural network remodeling has received special attention. In the ischemic penumbra, gliosis may have long-standing consequences for reperfused brain tissue [4, 5, 8, 13], as reactive astrocytes play key roles in neuroinflammation [9, 14–16]. With the development of MRI, it is now to longitudinally monitor the various cellular processes in and around the lesion areas [5]. Several studies visualized the cellular activity and dynamic pattern of structural changes by use of contrast agents with the MR system [4, 9, 17]. In this study, dynamic changes of reactive astrocytosis and neuronal connective pathway remodeling after transient cerebral ischemia were detected by MEMRI.

MEMRI, with the paramagnetic contrast agent Mn^2+^, can fulfill different aims by different administration methods to improve anatomical visualization. Mn^2+^ is an essential heavy metal that is needed for glutamine synthetase in astroglia and that is also a cofactor of superoxide dismutase [18, 19]. It has been reported that astrocytes act as “metal depots” and Mn^2+^ is primarily found in astroglia. At the same time, as a calcium analogue, Mn^2+^ can efficiently enter active neurons by voltage-gated calcium ion channels and be transported axonally and trans-synaptically along afferent and efferent connective pathways [4, 8, 9, 13, 20–22]. In MR imaging, Mn^2+^ can shorten the longitudinal relaxation time and transverse relaxation time, and because the MRI signal intensity is altered more in T1 than in T2, in vivo mapping of cellular activity and neuronal connections is facilitated [6].

Based on specific properties of Mn^2+^, reactive astroglia accelerate Mn^2+^ uptake and accumulation due to the high density of glial cells after stroke, resulting in T1 enhancement of the areas where astrocytes are mainly located [9]. Therefore, at the chronic stage, ring- or crescent-shaped enhancement was seen, consistent with a barrier established by astrocytosis along the boundary of the ischemic core. Astrocytes, as the most abundant subtypes of glial cells [1], have dual roles in CNS insults and are receiving more interest with regard to their beneficial and detrimental effects on surrounding cells and overall outcomes. Previous studies investigated their scar-forming property, which can cause chronic sequelae after CNS insults by inhibiting axonal regeneration and synaptic plasticity [23, 24]. However, in recent years, more positive roles of reactive astroglia have been discovered. As the main innate immune neuroglia in the CNS after injury, they can stimulate blood-brain barrier repair, counteract oedema, and may influence blood flow by regulating the blood vessel diameter and revascularization of blood capillaries to increase nutritional support of the nervous tissue [25]. Furthermore, astrocytes play key roles in the synaptic activity and regulation of neuronal circuitry [26, 27], as they are heavily involved in synaptogenesis, regulation of already formed neuronal connections and formation of new memories [26], and can even transdifferentiate into functional mature neurons [28, 29]. These findings suggest that reactive astroglia play significant roles in signaling pathways by affecting neurons and synapses. In our study combining in vivo imaging with post-mortem IF analysis, we detected the dynamic pattern of astrocytes and its positive temporal correlation with recovery of neuronal connective pathways.

At the same time, the loss and subsequent recovery of the manganese-induced T1 signal that we observed in the SN, which mostly receives indirect projections from the cortex, reflected the disrupted and subsequently restored connectivity of pathways by which Mn^2+^ was transferred [4, 21]. Other studies have reported the breakdown of axonal cytoskeletons and disruption of axonal transport [4, 30], which can explain the reduced manganese-related enhancement at the early stage in this study. Similarly, increased manganese enhancement at later stages demonstrated the recovery of the connective pathway. van der Zijden et al. showed the most significant loss of connectivity between the ipsilateral cortex and SN at 2 days after stroke. However, this was not present at later time points [4, 8]. Furthermore, an increasing number of studies using in vivo MRI detected white matter reorganization, including normalized T2, decreased fractional anisotropy (FA), and elevated mean kurtosis (MK) in subcortical lesion border zones at chronic time points, suggesting an improvement in white matter integrity [31]. These results point out that the connective pathway experiences degeneration and restoration, which can promote the uptake and transport of Mn^2+^. Hui et al. demonstrated a positive role of astrocytes in neuronal plasticity by combining the MK parameter with immunohistochemistry of GFAP [32, 33]. There were also studies showing that FA recovery or improvement was associated with astrocyte proliferation surrounding the lesion core [34, 35]. In this study, we performed systemic and stereotactic MEMRI to specifically track the cellular responses of astrocytes and neuronal pathways to detect the correlation between them.

Microglia/microphages did not contribute to systemic MEMRI enhancement because they remained in the lesion core. By IF, however, the morphological changes of microglia/macrophages, from round to amoeboid, were detected. This might also promote functional recovery based on evidence that amoeboid microglia play positive roles in brain inflammation modulation and neuroprotection by monitoring neural activity via transient contact with dendritic spines and synapses [36].

In our study, neurogenesis was not detected in the selected ROIs up to 18 days after stroke, which was not consistent with previous studies. In recent years, more studies have investigated the proliferation of neural stem cells (NSCs) at the subventricular zone (SVZ) and subgranular zone (SGZ) [37–40], where NSCs can produce new neurons in the adult brain. In addition to these well-known sites of neurogenesis, the circumventricular organs (CVOs) were confirmed to comprise a mid-line series of adult stem cell niches along the third and fourth ventricles. These NSCs possess the same characteristics as SVZ NSCs, which can produce mature neurons [41–43]. Other studies have found that astrocytes carry a latent neurogenic program when in the pathological state and that new reprogrammed neurons can form synaptic connections [28, 29]. In contrast, the number of NeuN (+) cells of the selected ROIs decreased in our observation time points. This may be attributed to the degree of ischemia and the time course and may need a longer observation period in subsequent studies.

Behavioral tests showed substantial functional recovery 7 days after stroke, in accordance with the demonstrated Mn-related signal enhancement of SN, proliferation of astrocytes, and morphological change of microglia. The data further corroborated the positive correlation between active astrogliosis and neuronal connective pathway remodeling.

In brief, inflammation is a major contributor to the pathophysiological process and is relevant to the degree of brain damage. Furthermore, accurate profiling of inflammatory events following transient cerebral ischemia is essential for patient rehabilitation [5]. In this study, by the in vivo imaging method, we detected a positive correlation between reactive astrogliosis and recovery of the connective pathways between the cortex and subcortical areas at the chronic stage. We also found the potential contribution of activated microglia/macrophages to the recovery of neuronal connectivity, although this needs further study for clarification.

## Conclusions

We demonstrated that reactive astroglia contribute to functional recovery after stroke by using an in vivo imaging method. The exact mechanism is still unknown, but this is the first study to combine systemic with stereotactic MEMRI to longitudinally observe the process of astrogliosis and its correlation with the neuronal connective pathway.

## Abbreviations

ANOVA, analysis of variance; BBB, blood-brain barrier; CVOs, circumventricular organs; ECA, external carotid artery; FA, fractional anisotropy; GFAP, glial fibrillary acidic protein; ICA, internal carotid artery; IF, immunofluorescence; MCA, middle cerebral artery; MCAO, middle cerebral artery occlusion; MEMRI, manganese-enhanced magnetic resonance imaging; MK, mean kurtosis; MRI, magnetic resonance imaging; NSCs, neural stem cells; SGZ, subgranular zone; SN, substantia nigra; STR, striatum; SVZ, subventricular zone

## Additional files

Additional file 1: Figure S1. Dose, concentration, and time dependence of brain enhancement on the manganese-enhanced MRI for STR. T1 MR images of normal rats at 24 h after administration of 47.1 μmol/kg of 33 mM MnCl_2_ solution (A1) and 125 μmol/kg of 50 mM MnCl_2_ solution (A2). T1 MR images of normal rats at 24 h, 2 and 4 days after administration of 267.9 μmol/kg of 50 mM MnCl_2_ solution (A3, A4, A5). T1 MR images of the day 3 stroke model at 1, 2, and 4 days after administration of 267.9 μmol/kg of 50 mM MnCl_2_ solution (B1, B2, B3). Temporal changes of the day 1, day 3, day 7, and day 14 stroke models at 24 h, 2 and 4 days after administration of 267.9 μmol/kg of 50 mM MnCl_2_ (C). ^*^ *P* < 0.05. (TIF 12843 kb) Additional file 2: Figure S2. HE staining of the lesion of STR. The HE staining of the lesion of STR at 1 day (A1), 3 days (B1), 7 days (C1), and 14 days after MCAO. The corresponding right panels present ×40 magnification images of HE staining (A2, B2, C2, D2). (TIF 2210 kb)
