# Supplementary figures and images for: Combining systemic and stereotactic MEMRI to detect the correlation between gliosis and neuronal connective pathway at the chronic stage after stroke
Source: J Neuroinflammation. 2016 Jun 18;13:156. doi: 10.1186/s12974-016-0622-7 (PMC4912752; doi:10.1186/s12974-016-0622-7)

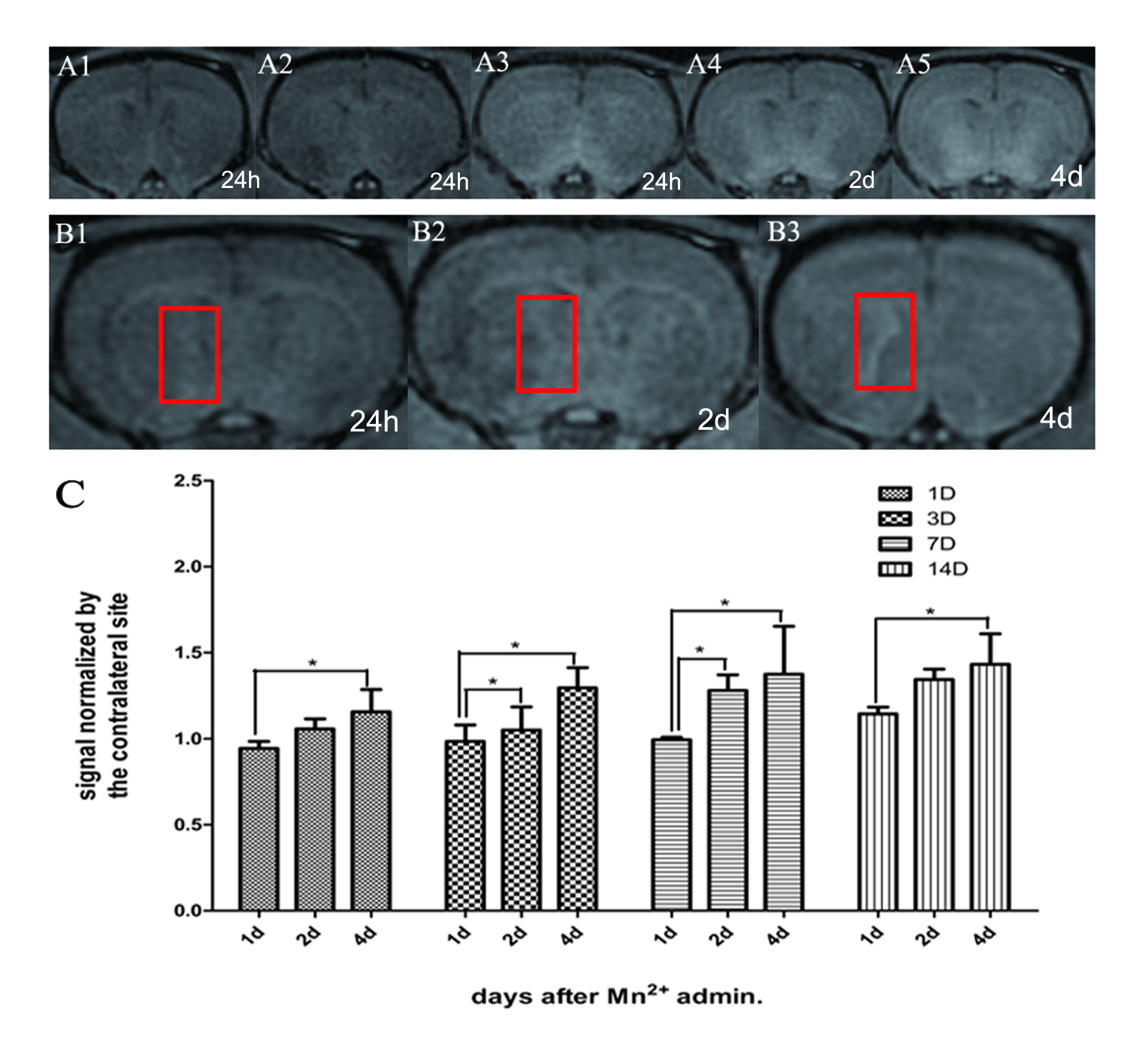

Supplement: Additional file 1: Figure S1. — Dose, concentration, and time dependence of brain enhancement on the manganese-enhanced MRI for STR. T1 MR images of normal rats at 24 h after administration of 47.1 μmol/kg of 33 mM MnCl2 solution (A1) and 125 μmol/kg of 50 mM MnCl2 solution (A2). T1 MR images of normal rats at 24 h, 2 and 4 days after administration of 267.9 μmol/kg of 50 mM MnCl2 solution (A3, A4, A5). T1 MR images of the day 3 stroke model at 1, 2, and 4 days after administration of 267.9 μmol/kg of 50 mM MnCl2 solution (B1, B2, B3). Temporal changes of the day 1, day 3, day 7, and day 14 stroke models at 24 h, 2 and 4 days after administration of 267.9 μmol/kg of 50 mM MnCl2 (C). * P < 0.05. (TIF 12843 kb) [file 12974_2016_622_MOESM1_ESM.tif]

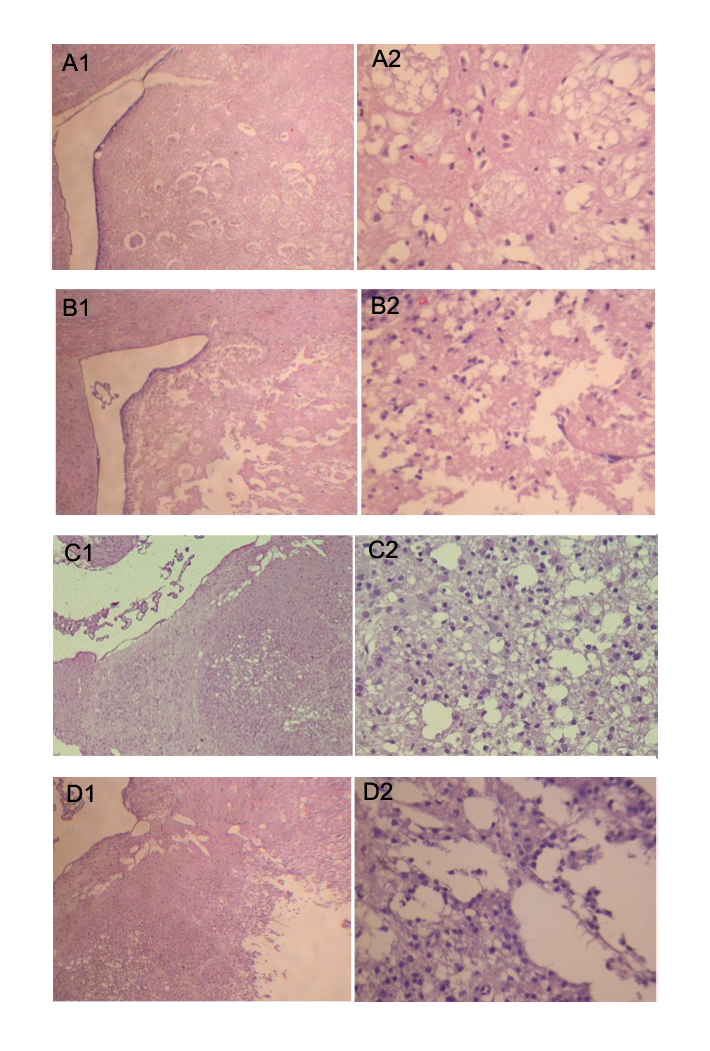

Supplement: Additional file 2: Figure S2. — HE staining of the lesion of STR. The HE staining of the lesion of STR at 1 day (A1), 3 days (B1), 7 days (C1), and 14 days after MCAO. The corresponding right panels present ×40 magnification images of HE staining (A2, B2, C2, D2). (TIF 2210 kb) [file 12974_2016_622_MOESM2_ESM.tif]
